# Identification of proteins from the secretory/excretory products (SEPs) of the branchiuran ectoparasite *Argulus foliaceus* (Linnaeus, 1758) reveals unique secreted proteins amongst haematophagous ecdysozoa

**DOI:** 10.1186/s13071-020-3964-z

**Published:** 2020-02-18

**Authors:** Aisha AmbuAli, Sean J. Monaghan, Kevin McLean, Neil F. Inglis, Michaël Bekaert, Stefanie Wehner, James E. Bron

**Affiliations:** 1 Institute of Aquaculture, School of Natural Sciences, University of Stirling, Stirling, FK9 4LA UK; 2 Department of Marine Science and Fisheries, College of Agricultural and Marine Sciences, Sultan Qaboos University, PO Box 34, 123 Al-Khoud, Sultanate of Oman; 3 Moredun Proteomics Facility, Moredun Research Institute, Pentland Science Park, Bush Loan, Penicuik, Midlothian, EH26 0PZ UK; 4 Max Planck Institute of Psychiatry, Kraepelinstr. 2-10, 80804 Munich, Germany

**Keywords:** Secretions, Immunomodulation, Fish lice, *Argulus*, Branchiura

## Abstract

**Background:**

It is hypothesised that being a blood-feeding ectoparasite, *Argulus foliaceus* (Linnaeus, 1758), uses similar mechanisms for digestion and host immune evasion to those used by other haematophagous ecdysozoa, including caligid copepods (e.g. sea louse). We recently described and characterised glands associated with the feeding appendages of *A. foliaceus* using histological techniques. The work described in the present study is the first undertaken with the objective of identifying and partially characterising the components secreted from these glands using a proteomic approach.

**Methods:**

*Argulus foliaceus* parasites were sampled from the skin of rainbow trout (*Oncorhynchus mykiss*), from Loch Fad on the Isle of Bute, Scotland, UK. The proteins from *A. foliaceus* secretory/excretory products (SEPs) were collected from the supernatant of artificial freshwater conditioned with active adult parasites (*n* = 5–9 per ml; *n* = 560 total). Proteins within the SEPs were identified and characterised using LC-ESI-MS/MS analysis. Data are available *via* ProteomeXchange with identifier PXD016226.

**Results:**

Data mining of a protein database translated from an *A. foliaceus* dataset using ProteinScape allowed identification of 27 predicted protein sequences from the *A. foliaceus* SEPs, each protein matching the criteria of 2 peptides with at least 4 contiguous amino acids. Nine proteins had no matching sequence through OmicsBox (Blast2GO) analysis searches suggesting that *Argulus* spp. may additionally have unique proteins present in their SEPs. SignalP 5.0 software, identified 13 proteins with a signal sequence suggestive of signal peptides and supportive of secreted proteins being identified. Notably, the functional characteristics of identified *A. foliaceus* proteins/domains have also been described from the salivary glands and saliva of other blood-feeding arthropods such as ticks. Identified proteins included: transporters, peroxidases, metalloproteases, proteases and serine protease inhibitors which are known to play roles in parasite immune evasion/induction (e.g. astacin), immunomodulation (e.g. serpin) and digestion (e.g. trypsin).

**Conclusions:**

To our knowledge, the present study represents the first proteomic analysis undertaken for SEPs from any branchiuran fish louse. Here we reveal possible functional roles of *A. foliaceus* SEPs in digestion and immunomodulation, with a number of protein families shared with other haematophagous ectoparasites. A number of apparently unique secreted proteins were identified compared to other haematophagous ecdysozoa.
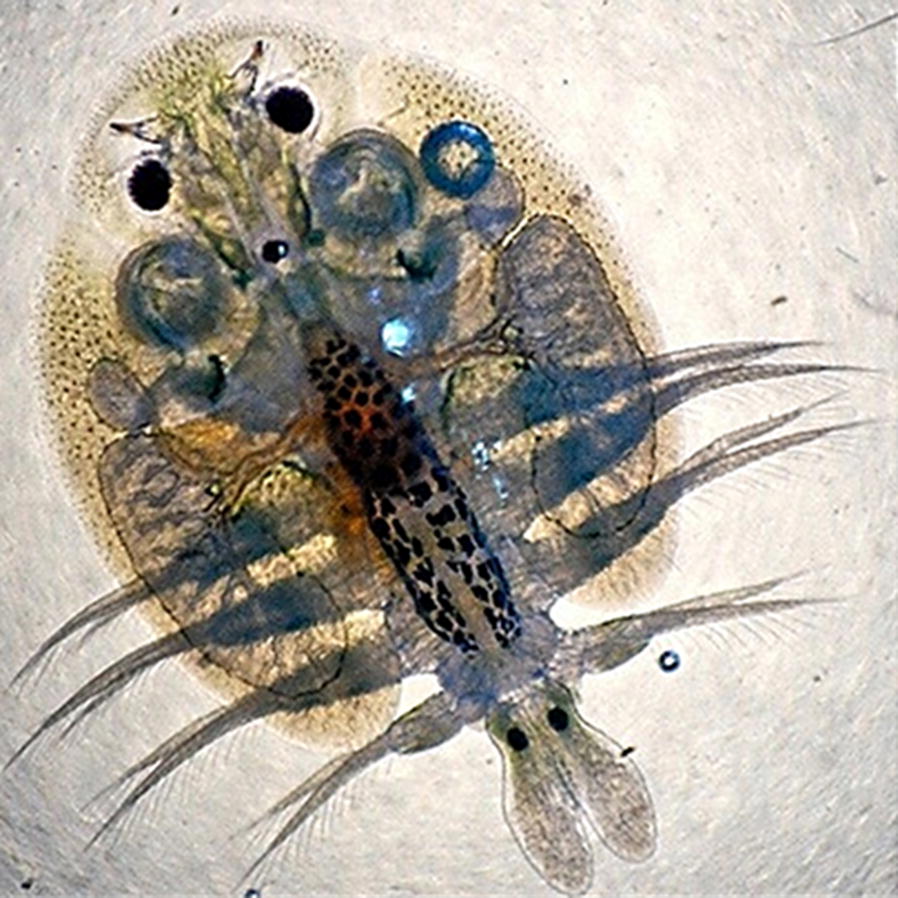

## Background

*Argulus foliaceus* (Linnaeus, 1758) is a member of the branchiuran family Argulidae and has a worldwide distribution [1–3]. In the UK, this parasitic arthropod causes the condition argulosis, which has economic impacts for both aquaculture and sports fishing industries [4] and affects wild and cultured fish populations [5, 6].

*Argulus* spp. attach to their hosts for extended periods using the first maxillae, which form hooks as larvae but are modified into suction discs as adults. It has been hypothesised that to enable long term host contact and feeding, *Argulus* spp., like other ectoparasitic arthropods such as ticks and salmon lice (*Lepeophtheirus salmonis* (Krøyer, 1837)), must secrete a range of pharmacologically active components. These components have been suggested to be produced in the parasite’s spinal, proboscis and labial glands and to be delivered through the pre-oral spine and the mouth tube to facilitate blood-feeding [7, 8]. *Argulus* spp. have been reported to feed on blood [9–11], mucus [12] and host skin [13] and thus have a presumed requirement to modulate host immunity. Pharmacologically active substances delivered in parasite secretions aid in modulating such defence mechanisms including those associated with pain, haemostasis, inflammation, complement activation and tissue repair [14–19]. However, to date there are limited genomic resources available for detailed investigation of this parasite. Furthermore, little formal data have been collected on either the composition or function of *Argulus* spp. secreted proteins or other active components [20].

During blood-feeding, other haematophagous arthropods inject the host with salivary products rich in digestive enzymes and other pharmacologically active proteins that have anti-haemostatic, anti-inflammatory and immunomodulatory properties. These salivary products help in counteracting the host immune response and wound recovery and hence facilitate feeding [21–28]. Recently, proteomics-based studies have contributed considerably to the identification of proteins from the salivary glands of a number of haematophagous insects including different species of mosquitoes i.e. *Anopheles stephensi* Liston, 1901, *A. campestris*-like, *Aedes aegypti* (Linnaeus in Hasselquist, 1762) and *Culex pipiens quinquefasciatus* Say, 1823. These studies have provided data relating to the functional roles of these proteins, and have facilitated their molecular and biological characterisation [21]. Recent transcriptomic and proteomic studies of salivary glands from different species of ticks have similarly identified a diverse range of active molecules/proteins that modify the hosts’ physiology [29–31]. Given the success of these studies in identifying active components of the saliva of other haematophagous arthropods, a proteomics approach may prove similarly productive to decipher the composition and role of *A. foliaceus* SEPs.

In two earlier studies, western blots of whole body extracts of *A. siamensis* (Wilson, 1926) [32] and *A. foliaceus* [33] revealed the presence of a number of immunodominant polypeptides recognised by immune rainbow trout (*Oncorhynchus mykiss* (Walbaum, 1792)) and rabbit serum. Notably, and despite phylogenetic distance, antigenic cross-reactivity between *A. foliaceus* and parasitic caligid copepod (*L. salmonis* and *Caligus elongatus* (von Nordmann, 1832)) antigens was demonstrated using serum antibodies raised against *A. foliaceus* in rainbow trout [20]. Both caligid sea lice and branchiuran freshwater lice feed on the fish epithelium, mucus and blood to some degree. To allow this, sea lice SEPs comprise a number of immunomodulatory proteins [34, 35] including metallopeptidases, serine proteinases and cysteine proteinases; previously identified in tick saliva [36]. Sea lice SEPs also contain anti-oxidant proteins identified in helminths [37], and serine endopeptidases, e.g. trypsins, identified in flies [38]. Whether the mechanisms involved in parasitism are similar between sea lice and other arthropods parasitising fish has yet to be established.

From the foregoing, the aim of the present study was to identify and characterise protein components of the SEPs produced by *A. foliaceus*. Characterisation of these proteins is key to establishing the biological function of branchiuran/*A. foliaceus* SEPs and can assist in identifying potential vaccine candidates or drug targets for the future development of more sustainable argulosis control strategies. To this end, proteomic data generated by LC-ESI-MS/MS were searched against a bespoke protein database assembled using proteins translated from an *A. foliaceus* transcriptomic dataset (submitted to the EBI with a project number PRJEB34947) and likely biological roles for discovered proteins ascribed.

## Methods

### Sample collection and incubation in artificial freshwater

Adult argulids were collected directly from fish hosts, rainbow trout, from Loch Fad on the Isle of Bute, Scotland, UK. A total of 560 adult male and female parasites were used. Between five and nine parasites (depending on the parasite sizes) were placed in 1.5 ml sterile Eppendorf tubes containing 1 ml of artificial freshwater, prepared as described by Klüttgen et al. [39]. Artificial freshwater without parasites was used as a negative control (50 × 1.5 ml sterile tubes of 1 ml of artificial freshwater). All the tubes were incubated at 10 °C overnight (18 h). The following day all parasites were alive and active and water samples expected to contain SEPs from *A. foliaceus* were pooled into 2 × 50 ml sterile Falcon tubes and the negative controls pooled into an additional 50 ml sterile tube. These samples and the control were rapidly chilled to − 70 °C and maintained frozen until used for sodium dodecyl sulphate-polyacrylamide gel electrophoresis (SDS-PAGE).

### SDS PAGE

Secretion/excretion samples and the negative control were thawed on ice and centrifuged at 4000×g for 30 min to concentrate proteins of > 3 kDa through 3 kDa cut-off centrifugal filters (Amicon® Ultra, Millipore, Cork, Ireland) prior to sample denaturation. The protein content of concentrated SEPs was measured using a Pierce Bicinchoninic acid (BCA) protein assay kit (Thermo Fisher Scientific, USA) according to the manufacturer’s instructions.

A dilution series of *A. foliaceus* SEP samples for SDS-PAGE analysis [40] was set up for different stains, one for silver stain and one for Coomassie stain. The concentrated sample (1:1) was combined with 2× SDS sample buffer (SDS reducing buffer; 2.5 ml 0.5 M Tris-HCL pH 6.8, 2 ml glycerol, 4 ml 10% SDS, 0.31 g dithiothreitol (DTT), 2 mg bromophenol blue and DW added to give 10 ml). Sequential 2-fold dilutions of 1:4 and 1:8 dilutions were then made with RNAse-free water. The samples were kept on ice then denatured at 100 °C in boiling water for 5 min then centrifuged (Micsolite, Thermo IEC) for 2 min at 16000×*g*. Five µl of 2–250 kDa mixed range pre-stained molecular weight markers (Precision Plus Protein™ Standards- Bio-Rad, Hemel Hempstead, UK) were used to estimate the size of proteins and loaded into two Precast 12-well Polyacrylamide gels (12% Mini-PROTEAN^®^ TGX™ Precast Protein Gels, Bio-Rad) followed by 15 µl of the samples in each well in descending order of sample concentration. The gels were run at 130 V for 75 min followed by washing with DDW for 5 min. After electrophoresis, fixed proteins were visualised with QC colloidal Coomassie stain (Bio-Rad) and silver stain using a silver staining kit (ProteoSilver ™-PROTSIL1-1KT, St. Louis, USA). The gel was scanned using a benchtop scanner (EPSON expression 1680 Pro) and kept at 4 °C in a sealed polyethylene bag with DDW until sent for gel and liquid chromatography electrospray ionisation tandem mass spectrometry (GeLC-ESI-MS/MS). The lane of the 1:2 sample dilution was selected for MS/MS analysis conducted at the Moredun Research Institute, Midlothian, UK.

### LC-ESI-MS/MS

The protein identifications were performed at The Moredun Proteomics Facility, Moredun Research Institute (Scotland, UK). The gel lane was excised and sliced horizontally from top to bottom to yield a series of 24 equal gel slices of 2.5 mm depth. Each of the resulting gel slices was then subjected to standard in-gel destaining, reduction, alkylation and trypsinolysis procedures [41]. Digests were transferred to low-protein-binding HPLC sample vials immediately prior to LC-ESI-MS/MS analysis. Liquid chromatography was performed using an Ultimate 3000 Nano-HPLC system (Dionex, Leeds, England) comprising a WPS-3000 well-plate micro auto sampler, an FLM-3000 flow manager and column compartment, a UVD-3000 UV detector, an LPG-3600 dual-gradient micropump and an SRD-3600 solvent rack controlled by Chromeleon™ chromatography software (Dionex). A micro-pump flow rate of 246 µl min^−1^ was used in combination with a cap-flow splitter cartridge, affording a 1/82 flow split and a final flow rate of 3 µl min^−1^ through a 5 cm × 200 µm ID monolithic reversed phase column (Dionex) maintained at 50 °C. Samples of 4 µl were applied to the column by direct injection. Peptides were eluted by the application of 15 min linear gradient from 8–45% solvent B (80% acetonitrile, 0.1% (v/v) formic acid) and directed through a 3 nl UV detector flow cell. LC was interfaced directly with a 3-D high capacity ion trap mass spectrometer (amaZon-ETD, Bruker Daltonics, Bremen, Germany) via a low-volume (50 µl min^−1^ maximum) stainless steel nebuliser (cat. no. G1946-20260; Agilent, Santa Clara, CA, USA) and ESI. Parameters for tandem MS analysis were based on those described previously [42].

### Database mining

The MS/MS data, formatted as Mascot Generic Format (mgf), was imported into ProteinScape™ V3.1 (Bruker Daltonics) proteomics data analysis software for downstream mining of a custom *Argulus* database. This custom database was constructed using translated proteins from the transcriptome dataset of *Argulus foliaceus* (in the absence of a full *Argulus* genome sequence) and comprised 60,257 protein sequences in total (the sequence data for the transcriptome were submitted to the EBI with a project number PRJEB34947 under the title “*De novo* transcriptome sequencing of branchiuran fish lice, *Argulus foliaceus* (Linnaeus, 1758) and *Argulus coregoni* (Thorell, 1865)”). *De novo* assembly was performed on the cleaned RNA-seq raw data using Trinity v2.1.1 [43]. The selection of the Trinity tool for final assembly was decided upon following a trial with other assembler software such as Velvet and ABySS whereby the use of Trinity was found to give higher numbers of more consistent reads. All the sample reads were merged into a single dataset for each species and the assembly was run. Then Transdecoder v2.0.1 [44] was used to find the coding region within the transcripts. Identification of the coding region transcripts gave open reading frames (ORFs) and amino acid sequences, to prepare the assembled dataset for annotation. Annotation was achieved by (i) BLAST v2.2.31 software [45, 46] using the uniprot/trembl-invertebrates database as query [47]; and (ii) Annotation HMMER v3.1b2 [48] using the Pfam A v29.0 as query [49].

Database searches were conducted utilising the Mascot™ V2.5.1 (Matrix Science) search engine. Mascot search parameters were set in accordance with published guidelines [50] and to this end, fixed (carbamidomethyl “C”) and variable (oxidation “M” and deamidation “N, Q”) modifications were selected along with peptide (MS) and secondary fragmentation (MS/MS) mass tolerance values of 0.5 Da whilst allowing for a single 13C isotope. Protein identifications obtained from each of the 24 individual gel slices were compiled using the “protein list compilation” feature within ProteinScape, which parses the data to remove redundancies. From the compiled protein lists individual identifications deemed significant by MASCOT (score > 35 which indicates *P*-value < 0.05) (Additional file 1: Table S1) were inspected manually and considered significant only if (i) two peptides were matched for each protein; (ii) peptides were represented by a sequence coverage of > 5%; and (iii) each matched peptide contained an unbroken “b” or “y” ion series represented by a minimum of four contiguous amino acid residues. The compilation of all gel slices and manual validation left a list of 27 proteins significantly identified by LC-ESI-MS/MS analysis from the custom transcriptome derived *Argulus* database.

### Functional analysis

To assign identity and function to the sequences of the proteins passing the criteria for significance following LC-ESI-MS/MS, the sequences were then searched using OmicsBox/ (Blast2Go) analysis. Searches were performed using the whole NCBI Blast and InterProscan for Protein function assignment, and then sequences of the proteins were finally blasted using BLAST P against NCBI ‘Metazoan’ database to get accession numbers with the most reliable hits. Proteins were then assigned into functional groups by searching the InterProScan databases and Gene ontology databases. Annotations from both searches were then merged. The Gene Ontology (GO) terms assigned to each protein were then used to construct pie charts based on biological process, cellular component and molecular function. The number of proteins and percentage were included with each GO term. Separate Pfam searches were conducted and SignalP 5.0 (http://www.cbs.dtu.dk/services/SignalP/) was used to predict the presence and location of signal peptide cleavage sites in amino acid sequences, which could inform of any associated secretory property of the protein. The mass spectrometry proteomics data have been deposited to the ProteomeXchange Consortium *via* the PRIDE partner repository with the dataset identifier PXD016226.

## Results

### Protein profiles in secretory/excretory products (SEPs) by SDS PAGE

**Fig. 1:**
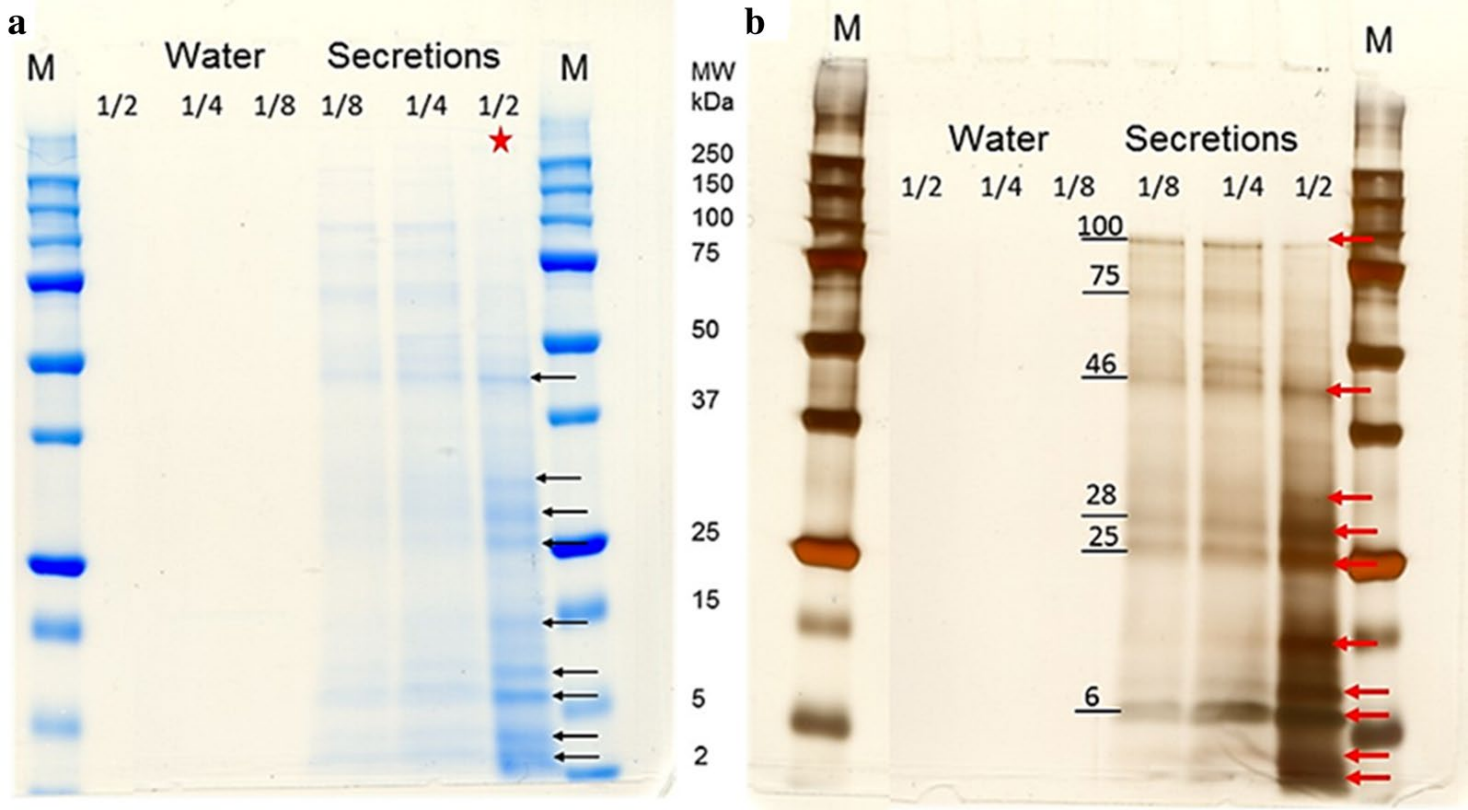
SDS-PAGE of *A. foliaceus* secretions. Secretory/excretory products (SEPs) were collected and proteins separated on 12% SDS-PAGE gels and stained with QC colloidal Coomassie stain (**a**) and silver stain (**b**) to visualise the protein bands. Numbers on the left indicate the approximate molecular mass (MW; kDa) of the proteins within the most diluted sample. Ten distinct bands (arrows) were observed after staining the gels. Molecular mass (2–250 kDa) marker is shown in the middle between the two gels. Asterisk indicates the lane from the Coomassie stained gel that was selected for GeLC-MS/MS analysis. No bands were seen in the water control

The protein content of harvested *A. foliaceus* SEPs was approximately 410 μg ml^−1^. SDS-PAGE analysis showed 10 intense bands of proteins with molecular masses in the range of 3–45 kDa. The use of three different dilutions of the secretions indicated a dilution effect in the intensity and number of bands obtained. The lack of bands observed from the sterilised artificial water (negative control) confirmed that the protein bands resulted from *A. foliaceus* parasites, either as secretions or excretory products, i.e. minimal environmental contamination (Fig. 1). Notably, there were 4 intense bands even in the most diluted sample; 1:8, with approximate molecular weights of 5, 25, 28 and 46 kDa, and an intense band was seen in the more dilute samples at around 100 kDa compared to the 1:2 diluted sample (Fig. 1b).

### LC-ESI-MS/MS analysis

SEPs were collected from 560 live and active *A. foliaceus* parasites of mixed sex adult life stages for GeLC-MS/MS analysis and protein identifications were confirmed by positive matches (Additional file 2: Table S2) with transcript sequences (unpublished data). From the compiled identified protein lists obtained from pooled *A. foliaceus* SEPs, 27 passed the specified “stringent” quality criteria and were associated with 27 annotated proteins (Tables 1, 2). To assign functional identity to these proteins, the amino acid sequences were searched against OmicsBox (Blast2GO) database and in order to find homologous metazoan proteins, the NCBI BLASTP program was used (Tables 1, 2) and the e-values of the proteins shown in the Table 1 were from the OmicsBox output. The InterProScan search assigned functional identity to 18 proteins, listed in Table 1, which shows Pfam functional descriptions, suggested functions from other arthropod studies and signal peptide predictions. The number of peptides, Signal P, molecular weight of proteins and percentage of sequence coverage are also listed (Table 1).

**Table 1:** List of proteins identified by GeLC-MS/MS analysis of *A. foliaceus* secretory/excretory products showing hits with OmicsBOx (Blast2Go) database

Family/Domain	SeqName	Description	MW (kDa)	No. Peptides	SC (%)	Pro. length	E-value	SignalP	Hits Organism	Function	NCBI ID
Vitellogenin_N	afol_3429.1p	Von Willebrand factor type D domain protein	172.5	55	39.4	1499	8.39E−11	Yes	*Ixodes scapularis*	Transporters, osmolality, immunity and clotting [25, 51–53]	XP_029822532.1
	afol_51180.1p	Vitellogenin-like isoform X1	183.8	44	30.1	1582	1.26E−11	No	*Ixodes scapularis*		XP_029826448.1
Hemocyanin	afol_43548.1p	Hemocyanin subunit type 1 precursor	81.2	18	26.3	697	0	Yes	*Argulus foliaceus*	Respiratory, protein storage [54]	CUH82791.1
	afol_52001.1p	Hemocyanin A chain	36.2	8	30.6	314	0	No	*Argulus foliaceus*		CUH82792.1
Astacin	afol_16671.1p	Protein SpAN-like	72.6	11	21.2	660	1.49E−55	Yes	*Saccoglossus kowalevskii*	Immunity; antifungal activity [55], food digestion, host penetration, immune evasion or activation [56–58]	XP_002739691.1
	afol_52344.2p	Protein SpAN-like	76.4	12	17.6	687	2.85E−50	No	*Penaeus vannamei*		XP_027221531.1
	afol_18725.8p	Blastula protease 10-like	50.7	8	18.4	462	1.44E−17	No	*Penaeus vannamei*		XP_027219989.1
Serpin	afol_27409.2p	Serpin B6-like	51.4	7	20.0	461	6.15E−66	No	*Rhipicephalus microplus*	Anticoagulation activity, modulate host immune response, regulation of host inflammation, antihaemostatic effects, and platelet aggregation [59–64]	AHC98669.1
	afol_25414.2p	Leukocyte elastase inhibitor-like	11.2	4	35.1	94	1.05E−16	No	*Ixodes scapularis*		EEC05896.1
Fasciclin	afol_20127.1p	Beta-ig-h3 fasciclin	37.1	5	23.1	333	1.64E−22	Yes	*Culex quinquefasciatus*	Mediate cell adhesion [65, 66]	XP_001847648.1
Trypsin	afol_3246.7p	Transmembrane protease serine 9	28.1	5	21.4	262	2.81E−12	No	*Penaeus vannamei*	Digestion, maintain host-parasite relationship [67]	XP_027224040.1
	afol_16878.20p	Trypsin-1	32.0	9	34.5	293	6.81E−61	Yes	*Ceratitis capitata*		XP_004520346.1
	afol_19181.1p	Serine protease 29	47.0	7	24.5	466	8.72E−87	Yes	*Bombus terrestris*		XP_020722765.1
	afol_18345.1p	Transmembrane protease serine 9-like	18.3	4	25.6	164	1.38E−17	No	*Drosophila navojoa*		XP_030246706.1
	afol_51127.2p	Trypsin	25.1	3	15.7	236	2.13E−57	No	*Penaeus vannamei*		ROT79324.1
	afol_56688.3p	Serine protease1/2	29.1	2	6.1	262	3.42E−39	Yes	*Ixodes scapularis*		XP_029851465.1
Peptidase_M14	afol_12392.3p	Mast cell carboxypeptidase A	48.3	5	16.7	424	4.21E−88	No	*Penaeus vannamei*	Proteolytic-enzyme [68])	XP_027226524.1
VIT	afol_32337.1p	Inter-alpha-trypsin inhibitor heavy chain H4-like isoform X2	98.2	4	7.4	883	2.31E−151	Yes	*Lingula anatina*	Proteinase inhibitor [36]	XP_013407760.1

*Note*: Description of suggested functions reported in different species of arthropods, and were blasted against Metazoa in NCBI BLASTP to obtain an Accession No. Signal P was used to predict the secretory property of the protein

*Abbreviations*: MW, molecular weight of protein; SC, sequence coverage; E-value, from the OmicsBox output

**Table 2:** Proteins identified from *A. foliaceus* secretory/excretory products by mass spectrometric analysis that showed no hits in OmicsBox/Blast2GO and were blasted against Metazoa in NCBI BLASTP, signal P

Protein ID	Protein length	MW (kDa)	SC (%)	E-value (BlastP)	NCBI ID	Signal P
afol_3444.2p	192	20.7	40.1			No
afol_55421.1p	140	14.9	42.1	0.57	XP_008192422.1	Yes
afol_9654.1p	408	44.7	16.2	5.4	XP_027221531.1	Yes
afol_25364.1p	438	48.9	14.4	0.24	XP_029189514.1	No
afol_15401.2p	230	25.7	13			No
afol_2470.1p	111	11.7	17.1	0.006	XP_017135474.1	No
afol_50565.1p	388	43.8	10.6			Yes
afol_52850.1p	399	43.7	6	0.083	XP_023212714.1	Yes
afol_45298.1p	189	21.5	25.4	0.49	EEC08850.1	Yes

*Abbreviations*: MW, molecular weight of protein; SC, sequence coverage; E-value, from the NCBI BlastP

From the initial list 9 proteins did not return any matches from OmicsBox (Blast2GO) and only 6 out of these 9 showed significant hits when blasted against Metazoa in NCBI BLASTP (Table 2).

Signal P analysis showed that out of these 27 proteins 13 were found to have a signal peptide sequence (Tables 1, 2).

### Assigned function of SE products

**Fig. 2:**
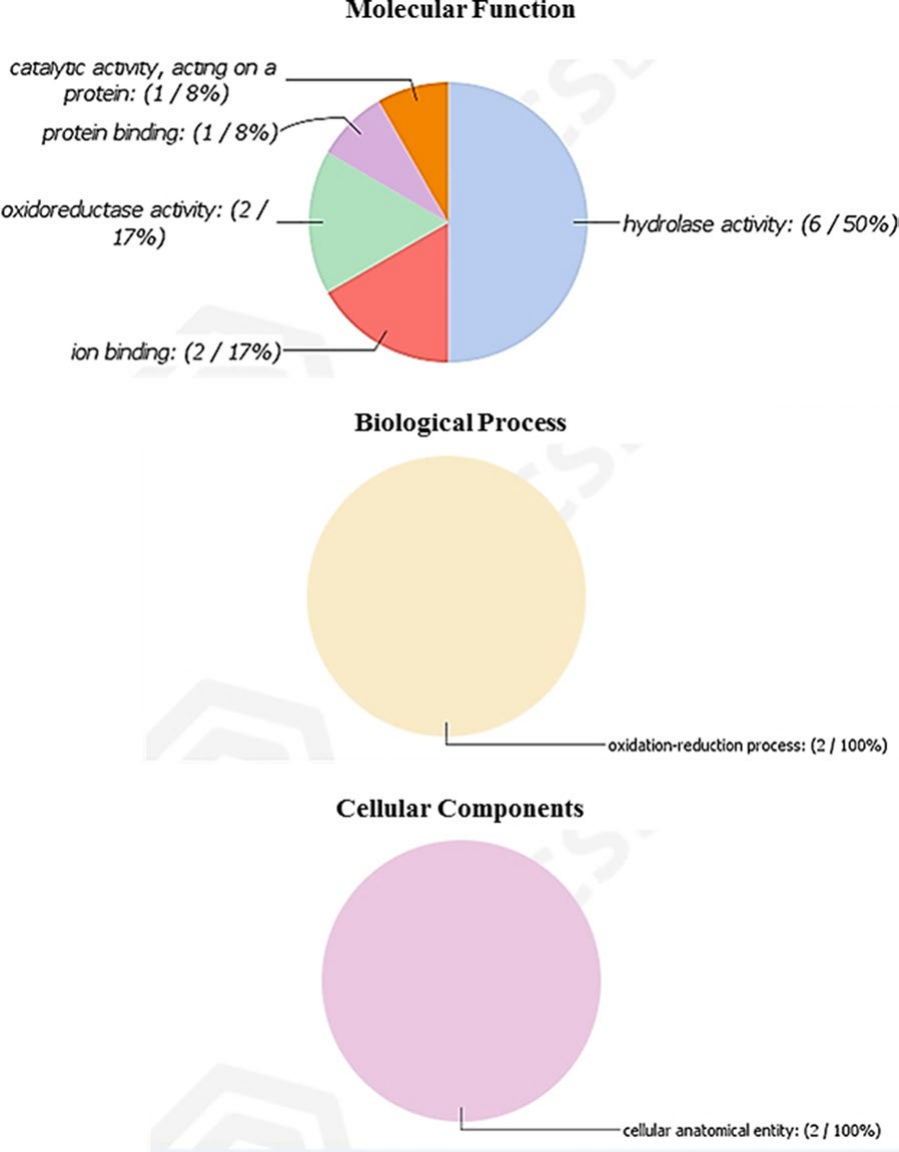
GO distribution of the proteins identified from *A. foliaceus* secretory/excretory products

Proteins identified from the SEPs were assigned GO terms within the biological process, cellular component and molecular function domains (Fig. 2). Proteins associated with molecular function accounted for 8% catalytic activity and 50% hydrolase activity. The most represented proteins in the biological process category (Fig. 2b) were assigned to oxidation-reduction processes. Cellular component represented only one GO term (with 2 entries), assigned to cellular anatomical entity (Fig. 2c) indicating the difficulty in characterising parasite SEPs in the absence of an annotated genome. OmicsBOx data (Additional file 3: Table S3) summarised the GO, InterProScan domain, families and IDs distributions data that resulted from OmicsBox hits of SE products, which showed functional data of the 27 identified proteins. This table represents the range of nominal roles assigned to the group of proteins found in SEPs of *A. foliaceus*.

## Discussion

Identifying the SEPs of *A. foliaceus* is important for determining how the parasite establishes host attachment and facilitates blood-feeding, as the components of ectoparasite secretions are known to play functional roles in such interactions [51–53]. In this regard, the secretions of *Argulus* spp. resemble those in the saliva of ticks and other haematophagous arthropods, containing bioactive molecules released to maintain a successful feeding site. Using LC-ESI-MS/MS, proteins such as serpin, trypsin and fascilin have been previously associated with this role in ticks (e.g. *Ornithodoros moubata* [25]), mosquitoes (e.g. *Anopheles culicifacies* [54]) and sea lice (e.g. *L. salmonis* [55]) saliva/salivary glands and SEPs. Sea lice trypsins, vitellogenin-like proteins and proteins with immunomodulatory functions or host adhesion properties have similarly been studied with suggestions of their potential as vaccine antigen candidates [56–58]. In order to feed, *Argulus* spp. doubtless needs to modulate host immune defence mechanisms (haemostasis and immunity) and inhibit host tissue repair responses in similar ways to other haematophagous arthropods such as ticks [59–62].

SDS-PAGE of the SEPs of *A. foliaceus* revealed a range of different protein bands between 3–100 kDa. Ruane et al. [33] showed similar, but more numerous, protein profile ranges from whole *A. foliaceus* homogenates, with proteins of molecular weights between 15–100 kDa; however, the authors suggested that the absence of higher molecular weight proteins may be due to the inability of the Coomassie stain to detect these potentially low abundance proteins in their study. From *A. siamensis* homogenates [32], protein bands between 16.22–130.55 kDa were detected by SDS-PAGE with intensely stained bands of > 66 kDa. In contrast to these previous studies, analysis in the present study was limited to *A. foliaceus* SEPs run under denaturing conditions, confirming the presence of secreted proteins, which were detectable in the most concentrated sample preparation (1:2 dilution) by Coomassie stain, mostly in the lower molecular weight range of 5–46 kDa. No proteomic studies on the secretions of any branchiuran including *Argulus* spp., had previously been conducted prior to this study, especially since genomic resources are limited. Therefore, in order to identify the major proteins of *A. foliaceus* SEPs, an integrated transcriptomic (manuscript in preparation) and proteomic approach was used. The (LC-ESI-MS/MS) data combined with the *A. foliaceus* transcriptome, used as a reference database, resulted in the identification of a number of different proteins nominally secreted by the parasite.

Overall, the analysis of the SEPs sample identified 27 proteins, of which only 18 were recognised by OmicsBox (Blast2GO) analysis and the other 9 protein sequences could not be recognised, and therefore could not be assigned a role at the present time. This is far fewer than the number of proteins identified from other parasite secretory products (e.g. 135 in the saliva of the hard tick *Haemaphysalis longicornis* [36] or 187 in the SEPs of *L. salmonis* [55]. Parasite SEP protein yields can be enhanced by inducing salivation or stimulation of the salivary glands using dopamine and pilocarpine, but with varying success [34, 36, 51, 55]. The overall number of identified proteins would also be anticipated to increase when a fully annotated genome becomes available. Some of the proteins that were identified are highly abundant in arthropods, particularly ecdysozoans, for example vitellogenins (vitellogenin-N) (e.g. in *L. salmonis* [63]) and haemocyanins (e.g. in crabs (*Cancer magister*) [64]) thus may simply be residual. Nonetheless, many of these proteins have previously been suggested to play a role in host infection in other haematophagous arthropods. These include metallopeptidases such as Peptidase_M14 and Astacin (Peptidase family M12A), proteases such as trypsin; and serpin; and other protein domains such as fasciclin and VIT (Vault protein inter-alpha-trypsin domain). Of the 27 identified proteins, 13 proteins carried a predicted signal sequence using Signal P suggesting them to be extracellular proteins and discharged within the secretions of the parasites. Identification of a number of signal peptides, short peptides (~ 16–30 aa) that direct newly synthesized proteins towards the secretory pathway [65], suggests that some of the proteins identified in the present study are secreted products, which notably included some vitellogenins and haemocyanins (Table 1). Such signal peptides target a protein for translocation across the endoplasmic reticulum (ER) membrane in eukaryotes [66]. The lack of a predicted signal peptide for other proteins discovered in this study may not indicate that they were not secreted but may simply result from the partial nature of the protein sequences, which were insufficient for prediction by Signal P; however, further studies need to confirm this.

This study identified a number of putative novel proteins (i.e. with no similarity in metazoan databases) from *A. foliaceus* secretions. Although the biological functions of these secreted proteins are unknown, they may have properties controlling physiological functions during *Argulus* attachment. As homologues for some of these proteins could not be found in the OmicsBox (Blast2GO) and NCBI databases, this could suggest that *Argulus* may have other unique proteins compared to other well characterised haematophagous ecdysozoa, such as insects, ticks and nematodes, for potentially modulating or evading their host’s immune system. However, considering the low SC% values and high e-values of a number of these proteins, their functional role is questionable at the current time. Other proteins, however, were analysed where functional identification was successful in this study and these have also previously been described in other haematophagous arthropods such as in tick salivary proteomes [36, 67, 68]. The role of this latter group of proteins from *A. foliaceus* secretions may therefore be similar to that played by their homologues in other ectoparasites during host-parasite interactions and supports their importance for *A. foliaceus* in feeding, digestion and evading host immune defences.

Vitellogenin is a lipoprotein generally related to reproduction in arthropods; however, it has been shown that the production of this protein can be positively associated with the size of blood meals, as is the case in ticks, where vitellogenin binds to the derivative haem from the host to initiate the reproduction cycle [69–71]. This was supported by the findings of Galay et al. [69] who showed that silencing of the secretory ferritin gene of the hard tick *H. longicornis* affected two vitellogenin genes. Moreover, Rosell-Davis & Coons [71] showed that onset of feeding initiates vitellogenin production.

Recent proteomic analysis of *L. salmonis* SEPs found a vitellogenin-like protein unique to adult females [55]. Dalvin et al. [63] examining *L. salmonis* did not observe any transcription of this protein in the ovary but they did localise the presence of these proteins in the haemolymph [63] indicating the protein is circulated through the louse and thus may have a role in reproductive processes following feeding similar to ticks. Further studies to localise this protein in *Argulus* spp. need to be conducted to give an indication of its reproductive or additional functional roles in this parasite.

Haemocyanin proteins in arthropods have been characterised as the main oxygen transporters in the haemolymph of many species [72]. In addition to their respiratory role, haemocyanin proteins are also involved in a range of other physiological processes including osmoregulation, protein storage and enzymatic activities [73, 74]. Destoumieux-Garzón et al. [75] revealed the importance of this protein to crustacean immunity in terms of the production of antifungal (poly) peptides. Recently, Pinnow et al. [72] identified two haemocyanin subunits from *A. foliaceus,* which were confirmed in this study, including two haemocyanin protein domains, hemocyanin subunit type 1 precursor and hemocyanin A chain. Although the production of haemocyanins is normal for respiration, Pinnow et al. [72] described haemocyanin 2 as a storage protein. Although apparently secreted proteins, i.e. possessing signal peptides, it should be noted, however, that both vitellogenin and haemocyanin could also be present as a result of damage to individual specimens and subsequent leakage of haemolymph. Therefore, the potential secretory roles of vitellogenin and haemocyanin proteins in *Argulus*, should they indeed be secreted, needs further investigation.

Metalloendopeptidase astacin and carboxypeptidase M14 identified in *A. foliaceus* SEPs have also been identified in the saliva of the haematophagous Mediterranean colubrariid (vampire) snail *Colubraria reticulata* which feeds on the blood of fishes [76] and also in *L. salmonis* [77]. Members of the astacin family have been considered to maintain blood flow to the lesion site through hydrolysis of fibrinogen and fibronectin [78, 79] leading to local haemorrhage [80]. It has been hypothesised that astacins have digestive and anticoagulation roles, also inactivating prey/host vasoactive peptides [76, 81] to maintain host-parasite relationships [82–85]. However, sea lice astacin is expressed in tegument glands associated with probable roles in lubrication of integument as opposed to feeding [86]. Therefore, the presence of these proteases in *Argulus* SEPs may be associated with functional roles in haematophagy, but as the feeding activity is similar to *L. salmonis*, they may also be involved in preventing drag when infecting moving fish.

Trypsins are proteases that have been found overexpressed in Atlantic salmon-fed *L. salmonis* lice (compared to lice feeding on less susceptible hosts) [77] and were also identified in *A. foliaceus* SEPs. Trypsins are secretory endopeptidases within the serine protease superfamily, known to facilitate food digestion, host penetration and to help in maintaining the host-parasite relationship [84]. They can also act as anticoagulating proteins [35, 76, 87, 88]. Although proteases have diverse biological functions within different tissues of blood-feeding arthropods [87], the detection of putative secreted trypsins in SEPs, verified by signal peptides, suggests that these trypsins have a vital role in *A. foliaceus* feeding processes and might play a role in parasite-host interactions. Trypsin-like serine proteases secreted in the salmon louse gut act as a general digestive protease [88, 89]. Trypsin-like proteases have been detected in the sea lice species *C. rogercresseyi* and *L. salmonis* and in their SEPs [35, 55, 77, 90, 91]. In addition, trypsin-like proteases have also been detected in the skin mucus of Atlantic salmon infected with *L. salmonis*, and have been suggested to play a role in facilitating feeding and evasion of the host immune response [92]. Whether the trypsins detected here are derived from salivary glands of *A. foliaceus* or simply gut contents requires further work.

Serpins, serine proteinase inhibitors, have previously been found to be secreted in arthropod saliva at the feeding site in order to facilitate blood meal acquisition through counteracting host defence mechanisms [36]. Two protein domains of serpin were identified in *A. foliaceus* SEPs. Serpin-mediated modulation of host immune response is achieved in ticks by impairing the hosts homeostatic and inflammatory responses, platelet aggregation and anticoagulation activity [24, 26, 54, 60, 93–98]. Salivary serpin 6 in the lone star tick, *Amblyomma americanum*, has been shown to have an inhibitory role in blood clotting and complement activation [60]. Moreover, *Ixodes scapularis* salivary serpin was found to inhibit the action of thrombin, platelet aggregation and trypsin at the tick-host interface [54]. Such serpins are likely to be inhibitors of pro-inflammatory and pro-coagulant proteases [99], such as *Iris2*, whereas serpins in *Ixodes ricinus* have been shown to inhibit inflammation by inhibiting cathepsin G and chymase [26]. Kim et al. [100] concluded that serpin was involved in host defence mechanisms during feeding by the inhibition of host trypsin and trypsin-like proteases. Weakened inflammatory responses have been reported recently in more susceptible carp species infected by *A. siamensis* [101] and suppression of pro-inflammatory responses by *L. salmonis* on infected Atlantic salmon is considered to contribute significantly to greater salmonid host susceptibility to salmon lice [102–104].

The presence of serpin in *A. foliaceus* secretions therefore implies a similar role in facilitating parasitism and modulating host immune responses in argulids. The observed presence of serpins by MS with a predicted MW of 51.4 kDa (afol_27409.2p; Table 1) is supported by SDS-PAGE of the SEPs sample, where an intense band was observed at 46.6 kDa. Similar to other blood-sucking arthropods these findings suggest that serpin in *Argulus* spp. may be one of the major components involved in evasion of the host defence mechanisms for ingestion of a successful blood meal. We recently used lectin-binding assays to characterise the *A. foliaceus* glands and have localised proteins with binding affinity to glycoaminoglycans (GAGs) to the spinal gland [7]. Serpins utilise GAGs for protease inhibition activity including modulation of coagulation [105] so it is likely that the spinal glands secretions are involved in *A. foliaceus* serpin activity.

Fasciclin protein domain from the *A. foliaceus* SEPs, also known as transforming growth factor-beta-induced protein [84], possessed a signal peptide. This protein has also been identified in the saliva of the argasid tick *Ornithodoros moubata* with the presence of signal peptides supporting a secretory nature [25], and salivary glands in the mosquito *Anopheles culicifacies* [106] and *L. salmonis* [77]. The fasciclin protein function was predicted to be associated with mediation of cell adhesion and signalling [106, 107], although its role in haematophagous arthropods remains unclear.

VIT (Vault protein inter-alpha-trypsin protein) domain was detected in the SEPs of *A. foliaceus* with an associated signal peptide, albeit with a relatively low SC% of 7.4 (Table 1). Interestingly, this protein domain has been identified recently, associated with von Willebrand factor type A protein domain, in the salivary subset of vampire snail *C. reticulata* (Mollusca: Gastropoda), feeding on fish blood. VIT has also been reported as the most overexpressed salivary transcript of the feeding-related proteins in the salivary glands of the parasitic snail [76]. This proteinase inhibitor was found, among several proteinase inhibitors, in the saliva of the hard tick *H. longicornis*, to be secreted into the feeding site to maintaining homeostasis, thus facilitate blood meal acquisition [36]. Due to the presence of VIT with a signal peptide in *A. foliaceus* SEPs, a similar mode of action may exist to that of *H. longicornis* VIT, but further validation is required.

In terms of molecular function, GO analysis was very restricted but revealed that the majority of the identified *A. foliaceus* proteins are nominally involved in catalytic activity acting on a protein (8%) and hydrolase activity (50%), and 17% in ion binding and oxireducatase activity, which were found also from *L. salmonis* adult SEPs [55]. Notably, in terms of biological function, oxidation-reduction processes accounted for 100% of the *A. foliaceus* SEPs identified, which may have roles in protection against host-derived reactive oxygen species [55, 108]. Proteins that were detected amongst the *A. foliaceus* SEPs but for which no biological function could be assigned on account of the absence of homologues in the Pfam and NCBI databases, remain of potential interest as the basis for further study.

The most common SE proteins assigned to molecular function were catalase, peptidases, hydrolases, endopeptidases, serine type endopeptidases, metallopeptidases and oxidoreductase. These proteinases were observed in both adult stages of *L. salmonis* in the study conducted by Hamilton et al. [55] and were suggested to potentially facilitate host-parasite interactions. For instance, serine peptidases and serine type endopeptidases may be of key importance to the success of the pre-adult- salmon louse in evading the host immune system before going into the final stage in the parasite life-cycle [109]. Catalase and other proteins detected in the saliva from the tick *H. longicornis* are suggested to play a role in detoxifying generated oxidants during blood meal acquisition and/or host oxidants associated with inflammation [36].

## Conclusions

To our knowledge, this study represents the first proteomic analysis undertaken for SEPs from any branchiuran fish louse. Here we reveal possible functional roles of *A. foliaceus* SEPs in digestion and immunomodulation, with a number of protein families shared with other haematophagous ectoparasites. A number of apparently unique secreted proteins were identified compared to other haematophagous ecdysozoa.

## Supplementary information

**Additional file 1: Table S1.** All the hits initially returned by the MS with highlights to those that fell below the MASCOT assigned score of 35 which indicates a *P*-value < 0.05, hits above 35 that did not meet validation criteria and proteins identified as positive as they pass all criteria.

**Additional file 2: Table S2.** *Argulus foliaceus* positive hits.

**Additional file 3: Table S3.** OmicsBOx (Blast2Go) analysis, using whole NCBI as Blast search and InterProscan for protein function assignment.

## Data Availability

The datasets which support the conclusions are included within the article. The mass spectrometry proteomics data have been deposited to the ProteomeXchange Consortium *via* the PRIDE [110] partner repository with the dataset identifier PXD016226 [111, 112].

## Abbreviations

LC-ESI-MS/MS: liquid chromatography-electrospray ionisation-tandem mass spectrometry
NaCl: sodium chloride
HPLC: high-performance liquid chromatography
BCA: bicinchoninic acid
kDa: kilodalton
EST: expressed sequence tag
SDSPAGE: sodium dodecyl sulphate polyacrylamide gel electrophoresis
PGE2: prostaglandin synthase E2
CaCl_2_: calcium chloride dihydrate
DW: distilled water
NaHCO_3_: sodium bicarbonate
VIT/VWA: Vault protein inter-alpha-trypsin domain and von Willebrand factor type A domain
PPIB: peptidyl-prolyl cis-trans isomerase B

## References

[1] Fryer G (1968). The parasitic Crustacea of African freshwater fishes: their biology and distribution. J Zool London..

[2] Kabata Z. Diseases of fish, book 1: crustacea as enemies of fishes. Snieszko SF, Axelrod H, editor. New Jersey: T.F.H Publications; 1970.

[3] Byrnes T (1985). Two new *Argulus* Species (Branchiura: Argulidae) found on Astralian bream (*Acanthopagurs* spp.). Aust Zool..

[4] Taylor NG, Sommerville C, Wootten R (2005). A review of *Argulus* spp. ocuring in UK freshwater.

[5] Rahman MM (1995). Some aspects of the biology of a freshwater fish parasite *Argulus foliaceus* (L.) (Argulidae, Branchiura, Crustacea). J Bangladesh J Zool..

[6] Sahoo P, Mohanty J, Garnayak SK, Mohanty BR, Kar B, Prasanth H (2013). Estimation of loss due to argulosis in carp culture ponds in India. Indian J Fish..

[7] AmbuAli A, Monaghan SJ, Al-Adawi K, Al-Kindi M, Bron JE (2019). Histological and histochemical characterisation of glands associated with the feeding appendages of *Argulus foliaceus* (Linnaeus, 1758). Parasitol Int..

[8] Øvergård A-C, Hamre LA, Harasimczuk E, Dalvin S, Nilsen F, Grotmol S (2016). Exocrine glands of *Lepeophtheirus salmonis* (Copepoda: Caligidae): Distribution, developmental appearance, and site of secretion. J Morphol..

[9] Hoffman GL (1977). *Argulus*, a branchiuran parasite of freshwater fishes.

[10] Mikheev VN, Mikheev AV, Pasternak AF, Valtonen ET (2000). Light-mediated host searching strategies in a fish ectoparasite, *Argulus foliaceus* L. (Crustacea: Branchiura). Parasitology..

[11] Walker P, Flik G, Bonga S, Gert F, Geert W (2004). The biology of parasites from the genus *Argulus* and a review of the interactions with its host. Host-parasite interactions.

[12] LaMarre E, Cochran PA (1992). Lack of host species selection by the exotic parasitic crustacean, *Argulus japonicus*. J Freshw Ecol..

[13] Van der Salm AL, Nolan DT, Spanings FAT, Wendelaar Bonga SE (2000). Effects of infection with the ectoparasite *Argulus japonicus* (Thiele) and administration of cortisol on cellular proliferation and apoptosis in the epidermis of common carp, *Cyprinus carpio* L., skin. J Fish Dis..

[14] Shimura S, Inoue K (1984). toxic effects of extract from the mouthparts of the *Argulus coregoni* Thorell. (Crustacea: Branchiura). Bull Jap Soc Sci Fish..

[15] Swanepoel JH, Avenant-Oldewage A (1992). Comments on the morphology of the pre-oral spine in *Argulus* (Crustacea: Branchiura). J Morphol..

[16] Gresty KA, Boxshall GA, Nagasawa K (1993). The fine structure and function of the cephalic appendages of the branchiuran parasite, *Argulus japonicus* Thiele. Philos Trans R Soc B Biol Sci..

[17] Møller OS, Olesen J (2010). The little-known *Dipteropeltis hirundo* Calman, 1912 (Crustacea, Branchiura): SEM investigations of paratype material in light of recent phylogenetic analyses. Exp Parasitol..

[18] Saha SK, Guha A, Banerjee A (2011). Feeding apparatus and associated glands in the freshwater fish ectoparasite *Argulus Siamensis* Wilson, 1926 (Branchiura). Crustaceana..

[19] Al-Darwesh AA, Al-Shabbani MAA, Faris BH (2014). Diagnostic and pathological study of *Argulus japonicas* in goldfish (*Carassius Auratus*). Glob J Bio-Sci Biotechnol..

[20] Von Reumont BM, Campbell LI, Jenner RA (2014). Quo vadis venomics? A roadmap to neglected venomous invertebrates. Toxins..

[21] Rawal R, Vijay S, Kadian K, Singh J, Pande V, Sharma A (2016). Towards a proteomic catalogue and differential annotation of salivary gland proteins in blood fed malaria vector *Anopheles culicifacies* by mass spectrometry. PLoS ONE..

[22] Carvalho-costa TM, Mendes MT, Vinicius M, Alvares T, Gomes M, Tiburcio S (2015). Immunosuppressive effects of *Amblyomma cajennense* tick saliva on murine bone marrow- derived dendritic cells. Parasit Vectors..

[23] Anstead CA, Korhonen PK, Young ND, Hall RS, Jex AR, Murali SC (2015). *Lucilia cuprina* genome unlocks parasitic fly biology to underpin future interventions. Nat Commun..

[24] Tirloni L, Seixas A, Mulenga A, Da Silva Vaz I, Termignoni C (2014). A family of serine protease inhibitors (serpins) in the cattle tick *Rhipicephalus* (Boophilus) *microplus*. Exp Parasitol..

[25] Díaz-Martín V, Manzano-Román R, Valero L, Oleaga A, Encinas-Grandes A, Pérez-Sánchez R (2013). An insight into the proteome of the saliva of the argasid tick *Ornithodoros moubata* reveals important differences in saliva protein composition between the sexes. J Proteomics..

[26] Chmelar J, Oliveira CJ, Rezacova P, Francischetti IMB, Kovarova Z, Pejler G (2011). A tick salivary protein targets cathepsin G and chymase and inhibits host inflammation and platelet aggregation. Blood..

[27] Weston-Davies W, Nuttall P (2002). Parasite saliva as a source of antiallergic agents. Lancet..

[28] Horn F, dos Santos PC, Termignoni C (2000). Boophilus microplus anticoagulant protein: an antithrombin inhibitor isolated from the cattle tick saliva. Arch Biochem Biophys.

[29] Ribeiro J, Anderson J, Manoukis N, Meng Z, Francischetti I (2011). A further insight into the sialome of the tropical bont tick, *Amblyomma variegatum*. BMC Genomics..

[30] Karim S, Singh P, Ribeiro JMC (2011). A deep insight into the sialotranscriptome of the gulf coast tick, *Amblyomma maculatum*. PLoS ONE..

[31] Francischetti I, Sa-Nunes A, Mans B, Santos I, Ribeiro J (2009). The role of saliva in tick feeding. Front Biosci..

[32] Saurabh S, Mohanty J, Garnayak SK, Sahoo PK (2012). Identification of immunodominant polypeptides of the freshwater fish lice *Argulus siamensis* (Wilson)—preliminary findings. Indian J Fish.

[33] Ruane N, McCarthy T, Reilly P (1995). Antibody response to crustacean ectoparasites in rainbow trout, *Oncorhynchus mykiss* (Walbaum), immunized with *Argulus foliaceus* L. antigen extract. J Fish Dis..

[34] Fast MD, Ross NW, Craft CA, Locke SJ, MacKinnon SL, Johnson SC (2004). *Lepeophtheirus salmonis*: characterization of prostaglandin E(2) in secretory products of the salmon louse by RP-HPLC and mass spectrometry. Exp Parasitol..

[35] Fast MD, Johnson SC, Eddy TD, Pinto D, Ross NW (2007). *Lepeophtheirus salmonis* secretory/excretory products and their effects on Atlantic salmon immune gene regulation. Parasite Immunol..

[36] Tirloni L, Islam MS, Kim TK, Diedrich JK, Yates JR, Pinto AFM (2015). Saliva from nymph and adult females of *Haemaphysalis longicornis*: a proteomic study. Parasit Vectors..

[37] Dzik JM (2006). Molecules released by helminth parasites involved in host colonization. Acta Biochim Pol..

[38] Boulard Chantal (1989). Degradation of bovine C3 by serine proteases from parasites *Hypoderma lineatum* (Diptera, Oestridae). Vet Immunol Immunopathol..

[39] Klüttgen B, Dülmer U, Engels M, Ratte H (1994). ADaM, an artificial freshwater for the culture of zooplankton. Water Res..

[40] Laemmli UK (1970). Cleavage of structural proteins during the assembly of the head of Bacteriophage T4. Nature..

[41] Shevchenko A, Jensen ON, Podtelejnikov AV, Sagliocco F, Wilm M, Vorm O (1996). Linking genome and proteome by mass spectrometry: large-scale identification of yeast proteins from two dimensional gels. Proc Natl Acad Sci USA.

[42] Batycka M, Inglis NF, Cook K, Adam A, Fraser-Pitt D, Smith DG (2006). Ultra-fast tandem mass spectrometry scanning combined with monolithic column liquid chromatography increases throughput in proteomic analysis. Rapid Commun Mass Spectrom..

[43] Grabherr MG, Haas BJ, Yassour M, Levin JZ, Thompson DA, Amit I (2015). Full-length transcriptome assembly from RNA-seq data without a reference genome. Nat Biotechnol..

[44] Haas BJ, Papanicolaou A, Yassour M, Grabherr M, Philip D, Bowden J (2013). Reference generation and analysis with Trinity. Nat Protoc..

[45] Altschul SF, Gish W, Miller W, Myers EW, Lipman DJ (1990). Basic local alignment search tool. J Mol Biol..

[46] Camacho C, Coulouris G, Avagyan V, Ma N, Papadopoulos J, Bealer K (2009). BLAST+: architecture and applications. BMC Bioinform.

[47] The UniProt Consortium UniProt (2015). The UniProt Consortium UniProt: a hub protein information. Acids Res..

[48] Eddy SR (1998). Profile Hidden Markov Models. Bioinformatics..

[49] Finn RD, Coggill P, Eberhardt RY, Eddy SR, Mistry J, Mitchell AL (2016). The Pfam protein families database: towards a more sustainable future. Nucleic Acids Res.

[50] Taylor GK, Goodlett DR (2005). Rules governing protein identification by mass spectrometry. Rapid Commun Mass Spectrom.

[51] Salát J, Paesen GC, Řezáčová P, Kotsyfakis M, Kovářová Z, Šanda M (2010). Crystal structure and functional characterization of an immunomodulatory salivary cystatin from the soft tick *Ornithodoros moubata*. Biochem J..

[52] Champagne DE, Wasserman HA, Kumar S, Singh S (2004). Pharmacological and immunological properties of saliva of the blood-feeding insects *Rhodnius prolixus* and *Aedes aegypti*. Physiol Entomol..

[53] Gillespie RD, Mbow ML, Titus RG (2000). The immunomodulatory factors of bloodfeeding arthropod saliva. Parasite Immunol..

[54] Ibelli AMG, Kim TK, Hill CC, Lewis LA, Bakshi M, Miller S (2014). A blood meal-induced *Ixodes scapularis* tick saliva serpin inhibits trypsin and thrombin, and interferes with platelet aggregation and blood clotting. Int J Parasitol..

[55] Hamilton S, McLean K, Monaghan SJ, McNair C, Inglis NF, McDonald H (2018). Characterisation of proteins in excretory/secretory products collected from salmon lice, *Lepeophtheirus salmonis*. Parasit Vectors..

[56] Johnson SC, Fast MD, Gert F, Geert W (2004). Interactions between sea lice and their hosts. Host-parasite interactions.

[57] Boxaspen K (2006). A review of the biology and genetics of sea lice. ICES J Mar Sci..

[58] Boxshall GA, Bravo S (2000). On the identity of the common *Caligus* (Copepoda: Siphonostomatoida: Caligidae) from salmonid netpen systems in southern Chile. Contrib Zool..

[59] Radulović ŽM, Kim TK, Porter LM, Sze S-H, Lewis L, Mulenga A (2014). A 24-48 h fed *Amblyomma americanum* tick saliva immuno-proteome. BMC Genomics..

[60] Mulenga A, Kim T, Ibelli AMG (2013). *Amblyomma americanum* tick saliva serine protease inhibitor 6 is a cross-class inhibitor of serine proteases and papain-like cysteine proteases that delays plasma clotting and inhibits platelet aggregation. Insect Mol Biol..

[61] Chmelar J, Calvo E, Pedra JHF, Francischetti IMB, Kotsyfakis M (2012). Tick salivary secretion as a source of antihemostatics. J Proteomics..

[62] Ribeiro JMC (1987). Role of saliva in blood-feeding by arthropods. Annu Rev Entomol..

[63] Dalvin S, Frost P, Loeffen P, Skern-Mauritzen R, Baban J, Rønnestad I (2011). Characterisation of two vitellogenins in the salmon louse *Lepeophtheirus salmonis*: molecular, functional and evolutional analysis. Dis Aquat Organ..

[64] Terwilliger NB, Ryan MC (2006). Functional and phylogenetic analyses of phenoloxidases from brachyuran (*Cancer magister*) and branchiopod (*Artemia franciscana*, *Triops longicaudatus*) Crustaceana. Biol Bull.

[65] Kapp K, Schrempf S, Lemberg MK, Dobberstein B, Zimmermann R (2009). Post-targeting functions of signal peptides. Protein Transp into endoplasmic reticulum.

[66] von Heijne G (1986). A new method for predicting signal sequence cleavage sites. Nucleic Acids Res..

[67] Tirloni L, Reck J, Terra RMS, Martins JR, Mulenga A, Sherman NE (2014). Proteomic analysis of cattle tick *Rhipicephalus* (*Boophilus*) *microplus* saliva: a comparison between partially and fully engorged females. PLoS ONE..

[68] Maritz-olivier C, Stutzer C, Jongejan F, Neitz AWH, Gaspar ARM (2007). Tick anti-hemostatics: targets for future vaccines and therapeutics. Trends Parasitol..

[69] Galay RL, Aung KM, Umemiya-Shirafuji R, Maeda H, Matsuo T, Kawaguchi H (2013). Multiple ferritins are vital to successful blood feeding and reproduction of the hard tick *Haemaphysalis longicornis*. J Exp Biol..

[70] Donohue KV, Khalil SMS, Sonenshine DE, Roe RM (2009). Heme-binding storage proteins in the Chelicerata. J Insect Physiol..

[71] Rosell-Davis R, Coons LB (1989). Relationship between feeding, mating, vitellogenin production and vitellogenesis in the tick *Dermacentor variabilis*. Exp Appl Acarol..

[72] Pinnow P, Fabrizius A, Pick C, Burmester T (2016). Identification and characterisation of hemocyanin of the fish louse *Argulus* (Crustacea: Branchiura). J Comp Physiol B..

[73] Paul RJ, Pirow R (1997). The physiological significance of respiratory proteins in invertebrates. Zool J..

[74] Depledge MH, Bjerregaard P (1989). Haemolymph protein composition and copper levels in decapod crustaceans. Helgoländer Meeresuntersuchungen..

[75] Destoumieux-Garzón D, Saulnier D, Garnier J, Jouffrey C, Bulet P, Bachère E (2001). Crustacean immunity: antifungal peptides are generated from the C terminus of shrimp hemocyanin in response to microbial challenge. J Biol Chem..

[76] Modica MV, Lombardo F, Franchini P, Oliverio M (2015). The venomous cocktail of the vampire snail *Colubraria reticulata* (Mollusca, Gastropoda). BMC Genomics..

[77] Braden LM, Sutherland BJG, Koop BF, Jones SRM (2017). Enhanced transcriptomic responses in the Pacific salmon louse *Lepeophtheirus salmonis* oncorhynchi to the non-native Atlantic Salmon *Salmo salar* suggests increased parasite fitness. BMC Genomics..

[78] Da Silveira RB, Wille ACM, Chaim OM, Appel MH, Silva DT, Franco CRC (2007). Identification, cloning, expression and functional characterization of an astacin-like metalloprotease toxin from *Loxosceles intermedia* (brown spider) venom. Biochem J..

[79] Da Silveira RB, Dos Santos Filho JF, Mangili OC, Veiga SS, Gremski W, Nader HB (2002). Identification of proteases in the extract of venom glands from brown spiders. Toxicon..

[80] Trevisan-Silva D, Gremski LH, Chaim OM, da Silveira RB, Meissner GO, Mangili OC (2010). Astacin-like metalloproteases are a gene family of toxins present in the venom of different species of the brown spider (genus *Loxosceles*). Biochimie..

[81] Lun HM, Mak CH, Ko RC (2003). Characterization and cloning of metallo-proteinase in the excretory/secretory products of the infective-stage larva of *Trichinella spiralis*. Parasitol Res..

[82] Bąska P, Wiśniewski M, Krzyżowska M, Długosz E, Zygner W, Górski P (2013). Molecular cloning and characterisation of *in vitro* immune response against astacin-like metalloprotease Ace-MTP-2 from *Ancylostoma ceylanicum*. Exp Parasitol..

[83] Park J-O, Pan J, Möhrlen F, Schupp M-O, Johnsen R, Baillie DL (2010). Characterization of the astacin family of metalloproteases in *C. elegans*. BMC Dev Biol..

[84] Kim HJ, Kim IS (2008). Transforming growth factor-β-induced gene product, as a novel ligand of integrin αMβ2, promotes monocytes adhesion, migration and chemotaxis. Int J Biochem Cell Biol..

[85] Gallego SG, Loukas A, Slade RW, Neva FA, Varatharajalu R, Nutman TB (2005). Identification of an astacin-like metallo-proteinase transcript from the infective larvae of *Strongyloides stercoralis*. Parasitol Int..

[86] Øvergård AC, Hamre LA, Harasimczuk E, Dalvin S, Nilsen F, Grotmol S (2016). Exocrine glands of *Lepeophtheirus salmonis* (Copepoda: Caligidae): distribution, developmental appearance, and site of secretion. J Morphol..

[87] Santiago PB, de Araújo CN, Motta FN, Praça YR, Charneau S, Bastos IMD (2017). Proteases of haematophagous arthropod vectors are involved in blood-feeding, yolk formation and immunity—a review. Parasit Vectors..

[88] Kvamme BO, Frost P, Nilsen F (2004). The cloning and characterisation of full-length trypsins from the salmon louse *Lepeophtheirus salmonis*. Mol Biochem Parasitol..

[89] Johnson SC, Ewart KV, Ross N, Murray HM (2002). Molecular cloning of trypsin cDNAs and trypsin gene expression in the salmon louse *Lepeophtheirus salmonis* (Copepoda: Caligidae). Parasitol Res..

[90] Valenzuela-Miranda D, Gallardo-Escárate C (2016). *Caligus rogercresseyi* serine proteases: Transcriptomic analysis in response to delousing drugs treatments. Aquaculture..

[91] Fast MD, Burka JF, Johnson SC, Ross NW (2003). Enzymes released from *Lepeophtheirus salmonis* in response to mucus from different salmonids. J Parasitol..

[92] Ross NW, Firth KJ, Wang A, Burka JF, Johnson SC (2000). Changes in hydrolytic enzyme activities of naive Atlantic salmon *Salmo salar* skin mucus due to infection with the salmon louse *Lepeophtheirus salmonis* and cortisol implantation. Dis Aquat Organ..

[93] Mudenda L, Pierlé SA, Turse JE, Scoles GA, Purvine SO, Nicora CD (2014). Proteomics informed by transcriptomics identifies novel secreted proteins in *Dermacentor andersoni* saliva. Int J Parasitol..

[94] Yu Y, Cao J, Zhou Y, Zhang H, Zhou J (2013). Isolation and characterization of two novel serpins from the tick *Rhipicephalus haemaphysaloides*. Ticks Tick Borne Dis..

[95] Prevot PP, Beschin A, Lins L, Beaufays J, Grosjean A, Bruys L (2009). Exosites mediate the anti-inflammatory effects of a multifunctional serpin from the saliva of the tick *Ixodes ricinus*. FEBS J..

[96] Prevot PP, Adam B, Boudjeltia KZ, Brossard M, Lins L, Cauchie P (2006). Anti-hemostatic effects of a serpin from the saliva of the tick *Ixodes ricinus*. J Biol Chem..

[97] Steen NA, Barker SC, Alewood PF (2006). Proteins in the saliva of the Ixodida (ticks): pharmacological features and biological significance. Toxicon.

[98] Leboulle G, Crippa M, Decrem Y, Mejri N, Brossard M, Bollen A (2002). Characterization of a novel salivary immunosuppressive protein from *Ixodes ricinus* ticks. J Biol Chem..

[99] Tirloni L, Kim TK, Coutinho ML, Ali A, Seixas A, Termignoni C (2016). The putative role of *Rhipicephalus microplus* salivary serpins in the tick-host relationship. Insect Biochem Mol Biol..

[100] Kim TK, Tirloni L, Radulovic Z, Lewis L, Bakshi M, Hill C (2015). Conserved *Amblyomma americanum* tick Serpin19, an inhibitor of blood clotting factors Xa and XIa, trypsin and plasmin, has anti-haemostatic functions. Int J Parasitol..

[101] Kar B, Moussa C, Mohapatra A, Mohanty J, Jayasankar P, Sahoo PK (2016). Variation in susceptibility pattern of fish to: do immune responses of host play a role?. Vet Parasitol..

[102] Johnson SC, Albright LJ (1992). Comparative susceptibility and histopathology of the response of naive Atlantic, chinook and coho salmon to experimental infection with *Lepeophtheirus salmonis* (Copepoda: Caligidae). Dis Aquat Organ..

[103] Fast MD (2014). Fish immune responses to parasitic copepod (namely sea lice) infection. Dev Comp Immunol..

[104] Braden LM, Barker DE, Koop BF, Jones SRM (2015). Differential modulation of resistance biomarkers in skin of juvenile and mature pink salmon, *Oncorhynchus gorbuscha* by the salmon louse, *Lepeophtheirus salmonis*. Fish Shellfish Immunol..

[105] Rein CM, Desai UR, Church FC (2011). Serpin-glycosaminoglycan interactions. Methods Enzymol..

[106] Sharma P, Sharma S, Mishra AK, Thomas T, De Das T, Rohilla SL (2015). Unraveling dual feeding associated molecular complexity of salivary glands in the mosquito *Anopheles culicifacies*. Biol Open..

[107] Gobert GN, Tran MH, Moertel L, Mulvenna J, Jones MK, McManus DP (2010). Transcriptional changes in *Schistosoma mansoni* during early schistosomula development and in the presence of erythrocytes. PLoS Negl Trop Dis..

[108] Robinson MW, Hutchinson AT, Dalton JP, Donnelly S (2010). Peroxiredoxin: a central player in immune modulation. Parasite Immunol.

[109] Firth KJ, Johnson SC, Ross NW (2000). Characterization of proteases in the skin mucus of Atlantic salmon (*Salmo salar*) infected with the salmon louse (*Lepeophtheirus salmonis*) and in whole-body louse homogenate. J Parasitol..

[110] Perez-Riverol Y, Csordas A, Bai J, Bernal-Llinares M, Hewapathirana S, Kundu DJ (2019). The PRIDE database and related tools and resources in 2019: improving support for quantification data. Nucleic Acids Res..

[111] Deutsch EW, Csordas A, Sun Z, Jarnuczak A, Perez-Riverol Y, Ternent T (2017). The ProteomeXchange consortium in 2017: supporting the cultural change in proteomics public data deposition. Nucleic Acids Res..

[112] Perez-Riverol Y, Xu QW, Wang R, Uszkoreit J, Griss J, Sanchez A (2016). PRIDE inspector toolsuite: Moving toward a universal visualization tool for proteomics data standard formats and quality assessment of proteomexchange datasets. Mol Cell Proteomics..

